# Determinants of continuum of care for maternal, newborn, and child health services in Ethiopia: Analysis of the modified composite coverage index using a quantile regression approach

**DOI:** 10.1371/journal.pone.0280629

**Published:** 2023-01-20

**Authors:** Aster Ferede Gebremedhin, Angela Dawson, Andrew Hayen

**Affiliations:** 1 Department of Public Health, College of Health Sciences, Debre Markos University, Debre Markos, Ethiopia; 2 School of Public Health, University of Technology Sydney, Sydney, Australia; American University of Beirut, LEBANON

## Abstract

Maternal and child mortality remain unacceptably high in the Sustainable Development Goals era. Continuum of care has become a key strategy for improving the health of mothers and newborns. Previous research on the continuum of care in Ethiopia is often limited to maternal health services. Maternal and child health services are inseparably linked, and an integrated approach to care is essential. This study assessed the continuum of maternal, newborn, and child health care and associated factors in Ethiopia. The analysis was based on the 2016 Ethiopian Demographic and Health Survey data. We restricted our analysis to women with their most recent children—alive and living with their mother- aged 12–23 months at the time of the survey (n  =  1891). The modified composite coverage index, constructed from twelve maternal and child health services, was calculated as an indicator of the continuum of care. Bivariable and multivariable quantile regression were used to analyse the relationship between the predictors and specific quantiles of the composite coverage index. The effect of each variable was examined at the 10th, 25th, 50th, 75th, and 95th quantiles. The results showed that the average composite coverage index value was 39%. The overall completion rate of the continuum of care was low (2%). Four % of the women did not receive any of the services along the continuum of care. Postnatal care for newborns had the lowest coverage (12%). This study provides evidence that factors such as the educational status of women, region, residence, socio-economic status, perceived distance to a health facility, pregnancy intention, mode of delivery, parity, and early antenatal care initiation influence the continuum of care differently across levels of the composite coverage index. The findings call for integrated and targeted strategies that aim to improve the continuum of care considering the determinants.

## Introduction

Maternal and child health (MCH) is an important public health issue [1]. Between 2000 and 2017, there was a 38 per cent reduction in the global maternal mortality ratio. However, pregnancy-related preventable morbidity and mortality remain unacceptably high, with many countries facing challenges to achieve the Sustainable Development Goals target of fewer than 70 maternal deaths per 100,000 live births. In 2017, about 295,000 maternal deaths occurred due to pregnancy and childbirth-related causes [2]. The WHO estimated that in 2019, 5.2 million children under five years died, mostly from preventable and treatable causes [3]. Maternal and child mortality disproportionately affects women and children in low and lower-middle-income countries. Sub-Saharan Africa lags behind all other regions globally in reducing child and maternal mortality [4,5]. While substantial progress has been made, the rate of maternal and child mortality in Ethiopia remains one of the highest in the world [2].

Effective coverage of MCH interventions has an enormous potential to avert poor health outcomes. MCH interventions are closely related and must be provided through a continuum of care (COC) approach [6]. The COC is touted as an important component of a strong health system needed to improve women’s and children’s health and survival [7]. The COC at its time dimension, denotes the continuation of care throughout the lifecycle and across levels of MCH service delivery [8]. Yet, there is no clearly defined and agreed upon measurement approach to assess it [9].

Previous studies that have investigated the COC have considered the key elements of MCH services; antenatal care (ANC), skilled birth attendance (SBA), and postnatal care (PNC) as separate entities [10–12]. Researchers have advocated using summary measures such as composite coverage index (CCI) that are better suited for providing an overall estimate of coverage based on combined coverage of several interventions [13,14]. The CCI is a comprehensive summary measure for reproductive, maternal, newborn, and child health (RMNCH) interventions derived from a weighted average of coverage of eight preventative and curative interventions. It gives equal weight to four stages in the COC: reproductive health, maternal and newborn care, immunisation, and management of child illness [15]. Previous studies have used modified CCI versions that use different measurement indicators [16–19]. A recent study assessed the COC by combining eleven essential RMNCH interventions [17]. The COC is influenced by a range of factors such. as individual and community characteristics [17,20–23], reproductive characteristics [18,24–28] and socio-economic characteristics [28–33].

The COC depicts a pathway from pre-pregnancy to postpartum and beyond, where each step adds value to ensure good pregnancy and child health outcomes. Understanding the COC is critical for developing and implementing effective strategies. In Ethiopia, completion of the maternal health COC is very low [34]. Previous research on the COC in Ethiopia is often narrow and focuses on maternal healthcare services [30,31,35,36]. MCH services are inseparably linked and an integrated approach to care is essential [37].

Much research has been conducted across countries on understanding COC and its associated factors [10,20,24,38–40]. However, the set of interventions used for the coverage measure is small and limited to selected maternal health interventions. In addition, the available studies have used analytical techniques that throw light on the mean of the outcome. Hence, their results do not give a complete picture of the relationship between explanatory and outcome variables.

Overall, there is increasing emphasis on the need to incorporate appropriate and broader sets of indicators in summary indices for assessing coverage comprehensively [15]. In this regard, our study used the recently modified CCI formula and examined the predictors of the COC for maternal, newborn, and child health services in Ethiopia. In addition, the present study contributes to the existing literature by using a quantile regression approach to assess the effects of the determinants along the entire distribution of the CCI.

## Methods

### Data source

This study was based on the 2016 Ethiopia Demographic and Health Survey (EDHS) conducted in Ethiopia. Ethiopia is structured into nine regional states namely Tigray, Afar, Amhara, Oromia, Somali, Benishangul-Gumuz, Southern Nations Nationalities and Peoples’ Region (SNNPR), Gambela, Harari and two city administrations, Addis Ababa and Diredawa. The EDHS provided data on a wide range of indicators relating to population, maternal, and child health issues at national and regional levels. The sampling frame used for the 2016 EDHS was a complete list of 84,915 enumeration areas (EAs) created for the 2007 Population and Housing Census. Samples were selected using two-stage stratified cluster sampling. First, 645 clusters or EAs (202 from urban and 443 from rural) were selected with probability proportional to EA size and with independent selection in each sampling stratum. Second, 28 households per cluster were selected with an equal probability systematic selection from the updated household list. A total of 15,683 women aged 15–49 and 12,688 men aged 15–59 were interviewed. The survey also included 10,641 children aged 0–5 from women who had given birth during the five years preceding the survey. The detailed information is presented in the 2016 EDHS report [41].

### Study population

According to the Expanded Programme for Immunisation in Ethiopia, children should receive BCG, polio, DPT (Diphtheria, Pertussis, Tetanus) and measles vaccinations before 12 months of age [42]. Because the DHS recodes the original data into different databases [43], we used the children’s recode with the intention to focus our study on children aged 12 to 23 months. Hence, out of the 10,641 women who had children under five years of age in the five years preceding the survey, 1820 women with their most recent children (alive and living with mother) aged 12–23 months were eligible. Eleven observations with missing values in receipt of care components were removed. After managing missing data, the analysis was restricted to a weighted sample of 1891 women (The unweighted sample size is 1809).

### Study variables

#### Outcome variable

The outcome variable was CCI, a composite metric consisting of a set of indicators representing interventions along the continuum. We used the modified CCI formula from a recent study by Oh J, et al. [17] which integrated Kerber et al.’s definition of the COC and WHO’s recommended MNCH care components [7]. The modified CCI is composed of essential MNCH interventions which are defined below:

$$\mathrm{Modified}\;\mathrm{CCI}=\frac{1}{6}\left(\frac{\mathrm{ANC}+\mathrm{TTN}}{2}+\frac{\mathrm{FD}+\mathrm{SBA}}{2}+\frac{\mathrm{PNCM}+\mathrm{PNCN}}{2}+\frac{\mathrm{BCG}+2\mathrm{DPT}3+2\mathrm{PL}+\mathrm{MSL}}{6}\mathrm{+AABF}+\mathrm{FP}\right)$$

ANC was defined as the use of at least four ANC visits by a woman during her pregnancy from a skilled provider. Birth protection against neonatal tetanus (TTN) was defined as whether or not women received two tetanus toxoid injections during pregnancy. Facility delivery (FD) was operationalised as delivered in a health facility by a skilled provider. Skilled birth attendance (SBA) indicates that births were delivered with the assistance of doctors, nurses/midwives, health officers, and health extension workers. To measure PNC for mothers (PNCM), we considered women who had a postnatal check during the first two days after birth. PNC for newborns (PNCN) was defined by newborns who had received a postnatal health check within the first two days after birth.

Family planning (FP) was defined as the percentage of women using any modern contraceptive method at the time of the survey. For the immunisation indicators, we assessed if a child took one dose of BCG vaccine, three doses of DPT-HepB-Hib (diphtheria, pertussis, tetanus- hepatitis B- haemophilus influenzae type B), three doses of polio vaccine (PL), and one dose of measles vaccine (MSL). Age-appropriate breastfeeding (AABF) denoted children who received breast milk and complementary foods. All indicators are equally weighted (except for the DPT and polio vaccines, which receive a weight of two because they require more than one dose. Women were asked whether they received each service, and their answers were coded ‘yes’ or ‘no’. This was followed by entering one for yes and zero for no into the modified CCI formula for each intervention use. Two categories of the calculated index were constructed: “1” for women receiving all MNCH interventions indicating full COC, and “0” for otherwise. To ascertain the internal consistency and validity of the items in relation to the underlying construct (CCI), Cronbach’s reliability coefficient was computed. Cronbach’s α reliability coefficient has a theoretical value of 0 to 1, and values greater than 0.7 are considered acceptable [44]. We found that Cronbach’s α reliability coefficient was 0.84 for the complete set of coverage indicators.

#### Independent variables

The independent variables included socio-economic and demographic factors (women’s age, marital status, women’s educational status, women’s employment status, region, residence—urban or rural- and wealth quintile), health service accessibility-related factors (health insurance membership, perceived distance from the health facility, media exposure), and obstetric characteristics (parity, caesarean delivery (CS), pregnancy intention, sex of the child, ANC initiation and history of pregnancy termination).

### Data analysis

The cleaned and recoded data were analysed using STATA 14. Frequencies and percentages were used to summarise the characteristics of variables. Data were presented using tables and graphs. We used quantile regression to analyse the relationship between the predictors and specific quantiles of the outcome variable (CCI). First introduced by Koenker and Bassett in 1978, quantile regression quantifies the association of explanatory variables with a conditional quantile of a dependent variable without assuming any specific conditional distribution [45]. Therefore, it models the quantiles instead of the mean as done in standard regression. In cases where the assumptions of mean regression are not met (i.e., linearity, homoscedasticity, independence, or normality), quantile regression can explain dependencies more accurately than classical methods [46]. Quantile regression provides a complete view of the effect of an independent variable on the outcome variable; therefore, it is possible to identify the more vulnerable groups and devise more effective interventions [47]. In this study, the distribution of CCI was found to be non-normal and the Breusch-Pagan test indicated the possible existence of heteroskedasticity (p  = 0.02), justifying the use of quantile regression.

First bivariable quantile regression was performed and variables with a P-value less than 0.05 in the bivariable analysis were entered into the multivariable quantile regression to identify the factors significantly associated with the outcome variable. The goodness of fit for the quantile regression model was indicated with pseudo-R^2^. A P-value less than 0.05 was considered statistically significant. The coefficient (b), standard error (SE) and 95% CI were estimated for the 10th, 25th, 50th (median), 75th, and 90th quantiles of CCI. Since the EDHS employed a complex sampling procedure, sampling weights were used in the analysis.

### Ethics

This study used publicly available data source and ethical approval was not required. The authorisation for using the data was granted from the DHS program. According to the EDHS 2016 report, all participant data were anonymised during the data collection [41].

## Results

### Socio-demographic characteristics

The median age of the mothers was 28 years (IQR 25,34). The majority (88.48%) of women lived in rural areas. Almost all (95%) were either married or in a union. About 55%, 63% and 25% of women were jobless, had no formal education and lived in the poorest wealth quintile, respectively. Other characteristics of the women are shown in Table 1.

**Table 1:** Background characteristics of the study population, Ethiopian Demographic Health Survey, 2016.

Variable	*n =* 1891	Percentage
**Women’s age** Median age = 28 years (IQR 25,34)		
15–24	466.4	24.7
25–34	989.6	52.3
35–49	434.8	23
**Marital status**		
Married/living with partner	1786.5	94.5
Not married /not in union	104.3	5.5
**Residence**		
Urban	217.9	11.5
Rural	1673	88.5
**Region**		
Tigray	145.9	7.7
Afar	19	1
Amhara	342.8	18.1
Oromia	836.0	44.2
Somali	66.7	3.5
Benishangul	20.3	1.1
SNNPR	393.4	20.8
Gambella	4.9	0.3
Metropolis	61.8	3.3
**Mother’s education**		
No education	1187	62.8
Primary	548.1	29.0
Secondary and higher	155.8	8.2
**Mother’s occupation**		
Not working	1037.3	54.9
Working	853.6	45.1
**Wealth index**		
Poorest	478.9	25.3
Poorer	376.6	19.9
Middle	414.1	21.9
Richer	342.7	18.1
Richest	278.6	14.7

Sixty-one per cent of the study participants had one to four pregnancies. Nearly 9% of the women had a history of termination of pregnancy. Additionally, 20% of the study participants had their first antenatal follow-up within 16 weeks of their pregnancy. Only 2% of women gave the most recent birth by CS. Most of the respondents (92%) reported having intended pregnancy. Approximately 96% of the women had no health insurance. About 60% of women perceived that the distance to a nearby health facility was a big problem. The majority (66%) of the study participants did not have media exposure (Table 2).

**Table 2:** Reproductive characteristics of the study population, Ethiopian Demographic Health Survey, 2016.

Variable	*n =* 1891	Percentage
**Sex of child**		
Male	874.9	46.3
Female	1016.0	53.7
**Parity**		
1–4	1148.2	60.7
5+	742.6	39.3
**Pregnancy intention**		
Intended	1731.1	91.6
Unintended	159.7	8.5
**Delivered by caesarean section**		
No	1847.3	97.7
Yes	43.6	2.3
**Ever terminated pregnancy**		
No	1726.7	91.3
Yes	164.2	8.7
**Attended ANC <4 months of pregnancy**		
No	1506.3	79.7
Yes	384.6	20.3
**Exposure to media (TV, radio, newspaper)**		
No	1253.4	66.3
Yes	637.4	33.7
**Health insurance covered**		
No	1811.3	95.8
Yes	79.5	4.2
**Distance to a health facility**		
Big problem	1130.7	59.8
Not a big problem	760.2	40.2

### Overall use of maternal and child health services

Among the mothers included in the study 623 (33%) had four or more ANC visits and 787 (42%) received two doses of tetanus injection. Women who delivered in a health facility were 647 (34%). Six hundred seventy-one women (36%) were attended by a skilled health provider.

**Fig 1:**
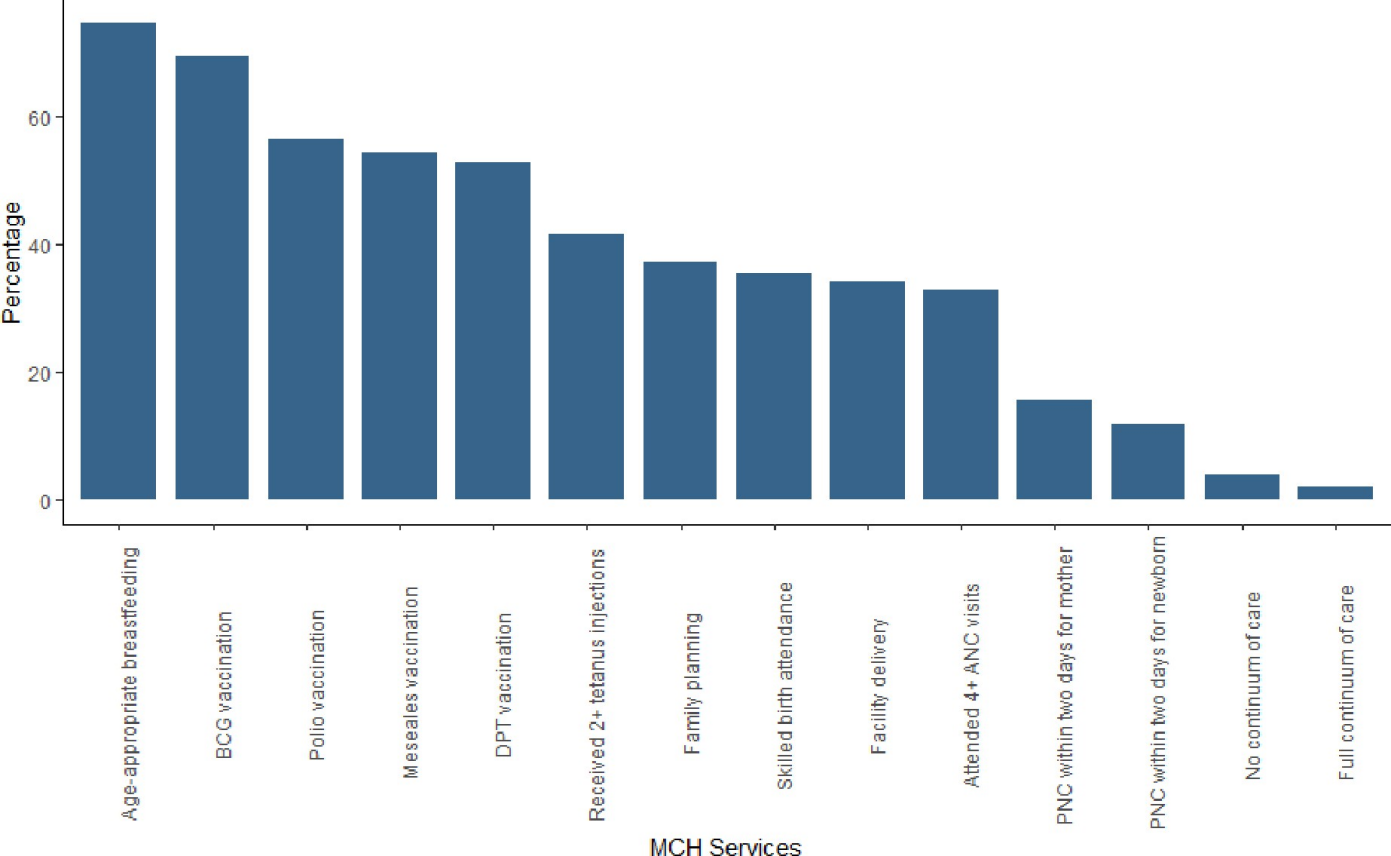
Components of the continuum of care (weighted n = 1891).

For postnatal care, 298 (16%) women received a health checkup within two days of delivery while 223 (12%) newborns had a postnatal check within two days. Seven hundred four women (37%) used modern contraceptive methods. Among the four vaccinations, 1313 (69%), 1001 (53%), 1027 (54%) & 1067 (56%) women had children who received BCG, three doses of DPT, measles, and three doses of polio respectively. A total of 1408 (75%) children received age-appropriate breastfeeding (Fig 1).

### Composite coverage index

As an indicator of COC, the CCI was computed for each mother. The average CCI value was 39% (Median = 0.39, IQR 0.22, 0.58). Among all the sampled women, 40 (2%) had the full range of services for the COC, while 73 (4%) women did not receive any of the twelve services.

### Determinants of the continuum of care

The bivariate analysis demonstrated that educational status, occupation, wealth index, residence, region, health facility distance, parity, pregnancy intention, caesarean delivery, attending ANC in less than four months of pregnancy and media exposure were associated with CCI. These variables were considered in the multivariable quantile regression analysis (see Table 3).

**Table 3:** The results of the multivariable quantile regression analysis.

	Quantile regression coefficients at different quantiles				
Variables	Quantile regression at 0.1 quantile (se)	Quantile regression at 0.25 quantile (se)	Quantile regression at 0.50 quantile (se)	Quantile regression at 0.75 quantile (se)	Quantile regression at 0.9 quantile (se)
Educational status					
Primary education	0.0455***	0.0333*	0.0833***	0.0556***	0.0648**
	(0.0174)	(0.0197)	(0.0282)	(0.0194)	(0.0289)
Secondary or more	0.199***	0.128***	0.117***	0.0556	0.0370
	(0.0605)	(0.0277)	(0.0293)	(0.0352)	(0.0400)
Occupational status					
Working	-0.0126	-0.00556	0.0222	0.0556***	0.0370
	(0.0145)	(0.0144)	(0.0170)	(0.0105)	(0.0227)
Wealth quintile					
Poorest	-0.0505	-0.0333	-0.122***	-0.139***	-0.139**
	(0.0370)	(0.0342)	(0.0426)	(0.0410)	(0.0660)
Poorer	-0.0152	-0.0167	-0.0222	-0.0278	-0.0247
	(0.0331)	(0.0347)	(0.0474)	(0.0342)	(0.0770)
Middle	0.0202	0.0111	-0.0333	-0.0278	-0.00617
	(0.0358)	(0.0355)	(0.0443)	(0.0334)	(0.0773)
Richer	0.00253	0.0278	0.0111	0.0278	0.0432
	(0.0385)	(0.0365)	(0.0446)	(0.0318)	(0.0659)
Perceived distance to a facility					
Distance not a big problem	0.0379***	0.0444***	0.0222	0.0278	0.0556**
	(0.0142)	(0.0150)	(0.0194)	(0.0207)	(0.0249)
Parity					
Parity 5+	0.0177	-9.93e-09	-0.0278	-0.0556***	-0.0463
	(0.0148)	(0.0147)	(0.0179)	(0.0188)	(0.0305)
Pregnancy intention					
Unintended pregnancy	-0.116***	-0.0444**	-0.00556	-0.0556*	-0.0432
	(0.0227)	(0.0218)	(0.0329)	(0.0286)	(0.0899)
Mode of delivery					
CS delivery	0.124**	0.0722	0.133*	0.167***	0.167***
	(0.0580)	(0.0579)	(0.0753)	(0.0572)	(0.0501)
ANC visit					
ANC within 4 months	0.129***	0.144***	0.139***	0.111***	0.102***
	(0.0304)	(0.0162)	(0.0191)	(0.0182)	(0.0259)
Region					
Tigray	0.136***	0.0722*	0.128***	0.139***	0.105***
	(0.0494)	(0.0386)	(0.0262)	(0.0307)	(0.0315)
Afar	-0.109**	-0.261***	-0.122***	-0.139***	-0.154***
	(0.0515)	(0.0393)	(0.0347)	(0.0313)	(0.0381)
Amhara	0.00758	-0.0833*	-7.45e-09	-0.0278	-0.0556
	(0.0515)	(0.0430)	(0.0337)	(0.0262)	(0.0429)
Oromia	-0.0126	-0.111***	-0.0444	-0.0556*	-0.0741**
	(0.0532)	(0.0400)	(0.0291)	(0.0292)	(0.0363)
Somali	-0.126**	-0.256***	-0.128***	-0.139***	-0.173***
	(0.0528)	(0.0400)	(0.0357)	(0.0303)	(0.0359)
Benishangul	0.0455	-0.0333	0.0444	0.0556**	-5.96e-08
	(0.0577)	(0.0458)	(0.0369)	(0.0277)	(0.0445)
SNNPR	-0.0404	-0.0944**	0.00556	0.0556*	0.0340
	(0.0491)	(0.0415)	(0.0321)	(0.0311)	(0.0365)
Gambella	-0.0808*	-0.122***	-0.0556*	0.0278	-0.0309
	(0.0433)	(0.0385)	(0.0314)	(0.0244)	(0.0317)
Residence					
Rural residence	-0.111***	-0.111***	-0.144***	-0.139***	-0.0895*
	(0.0399)	(0.0376)	(0.0442)	(0.0391)	(0.0495)
Media exposure					
Has media exposure	-0.00758	0.0167	0.0222	1.49e-08	-0.00309
	(0.0200)	(0.0174)	(0.0229)	(0.0188)	(0.0349)
Constant	0.242***	0.406***	0.511***	0.667***	0.781***
	(0.0550)	(0.0442)	(0.0388)	(0.0359)	(0.0512)
Observations	1,809	1,809	1,809	1,809	1,809
Pseudo R2	0.1734	0.2201	0.2506	0.2516	0.2126

Robust standard errors in parentheses.

*** p<0.01

** p<0.05

* p<0.1.

Multivariable analysis demonstrated that educational status had differential effects at different quantiles of the CCI. Mothers who completed primary education tended to have a significantly higher level of COC, as evidenced in the 10^th^ (β = 0.05, *P* = 0.009), 50^th^ (β = 0.08, *P* = 0.003), 75^th^ (β = 0.06, *P* = 0.004), and 90^th^ quantile (β = 0.06, *P* = 0.025) as compared with those mothers who had no formal education. Women who completed secondary education or above also had increased levels of COC compared to women who had no formal education at the 10^th^ (β = 0.199, *P* = 0.001), 25^th^ (β = 0.13, *P* <0.001), and 50^th^ quantiles (β = 0.12, *P* < 0.001).

Region of residence was also shown to variably affect CCI across different quantiles. At the 10^th^ quantile, residence in Tigray (β = 0.14, *P* = 0.006) has a positive association with CCI, showing a high COC as compared to residence in the metropolis regions (referring to Addis Ababa, Harari and Diredawa). In contrast, residence in Afar (β = -0.11, *P* = 0.035) and Somali (β = -0.13, *P* = 0.017) was negatively related to CCI. Similarly, living in Tigray (β = 0.13, *P* < 0.001), Afar (β = -0.12, *P* < 0.001) and Somali (β = -0.13, *P* < 0.001) were significantly associated with COC, as compared to living in the metropolis, at the 50^th^ quantile. The CCI level of the 25% quantile was negatively associated with residence in Afar (β = −0.26, *P* < 0.001), Oromia (β = -0.11, *P* = 0.006), Somali (β = −0.26, *P* < 0.001), SNNPR (β = -0.09, *P* = 0.023), and Gambella (β = -0.12, *P* = 0.002) as compared to a residence in the metropolis. At the 75^th^ quantile, women residing in Tigray (β = 0.14, *P* < 0.001) and Benishangul (β = 0.06, *P* = 0.045) had a high level of COC than women residing in the metropolis. In contrast, women residing in Afar (β = -0.14, *P* <0.001) and Somali (β = - 0.14, *P* < 0.001) had lower levels of COC compared with women residing in the metropolis. At the 90^th^ quantile, women who lived in Tigray (β = 0.10, *P* = 0.001) had a high level of COC relative to people who lived in the metropolis. Conversely, women who lived in Afar (β = -0.15, *P* < 0.001), Oromia (β = -0.07, *P* = 0.042) and Somali (β = -0.17, *P* < 0.001) had lower levels of COC compared with women from the metropolis.

The wealth quintile showed significant associations at the intermediate and higher quantiles. Women in the poorest quintiles had a lower COC at the 50^th^ (β = -0.12, *P* = 0.004), 75^th^ (β = -0.14, *P* = 0.001), and 90^th^ quantiles (β = -0.14, *P* = 0.035) than women in the richest quintiles. Significant negative associations between rural residence and continuum-of-care were observed at the 10^th^ (β = -0.11, *P* = 0.005), 25^th^ (β = -0.11, *P* = 0.003), 50^th^ (β = -0.14, *P* = 0.001), and 75^th^ (β = -0.14, *P* <0.001) quantiles of CCI. Occupational status was significant only at the 75^th^ quantile, where women who were working had significantly higher levels of CCI compared to their counterparts (β = 0.06, *p* < 0.001).

Regarding reproductive characteristics, attending ANC in less than four months of pregnancy was positively associated with a higher COC across all quantiles (10%: β = 0.13, *P* < 0.001; 25%: β = 0.14, *P* < 0.001; 50%: β = 0.14, *P* < 0.001; 75%: β = 0.11, *P* < 0.001; 90%: β = 0.10, *P* < 0.001). Moreover, delivery by CS increased COC and the increase was significant at the 10^th^ (β = 0.12, *P* = 0.033), 75^th^ (β = 0.17, *P* = 0.004), and 90^th (^β = 0.17, *P* = 0.001) quantiles. Compared to women who perceived distance as a significant problem, women who perceived distance to the nearby health facility was not a significant barrier were significantly associated with high levels of the COC at the lowest and highest quantiles. The estimates at the 10^th^, 25^th^, and 90^th^ quantiles were β = 0.04; *P* = 0.008, β = 0.04; *P* = 0.003, and β = 0.06; *P* = 0.026, respectively. The association between pregnancy intention and CCI was maintained only in the lower quantiles. Unintended pregnancy was significantly associated with lower CCI levels at the 10^th^ (β = -0.12, *P*<0.001) and 25^th^ (β = -0.04, *P* = 0.042) quantiles. Parity was significant only at the 75^th^ quantile. Women with a parity of five or more had significantly lower levels of receiving the elements of the COC compared to women of less than five parity (β = - 0.06, *P* = 0.003).

## Discussion

The COC has become a core principle to underpin strategies to save the lives of mothers and babies and promote health. An effective COC connects essential MNCH packages throughout adolescence, pregnancy, childbirth, postnatal periods, and into childhood [48]. This study assessed the COC and its predictors in Ethiopia using a quantile regression approach.

The finding of this study showed that the average CCI was 39%. A previous study reported higher estimates of average CCI for Ethiopia, ranging from 45% to 51% [19]. Similarly, another study from the Lao People’s Democratic Republic reported a higher estimate of average CCI [18]. The possible explanation for the variation could be the difference in the MCH coverage indicators incorporated in the summary indices. Our study finds that the overall completion of the COC was two per cent which was lower than that reported in the previous studies [12,27,30,36]. This variation could be explained by the difference in the measurement of the COC as we used the recently modified and more comprehensive coverage index [17].

Our study revealed that postnatal care for newborns has the lowest coverage along the continuum. In line with this finding, a study documented that coverage levels are particularly low around the time of birth and postnatal care consistently has among the lowest coverage of interventions on the COC [49]. Even though most maternal and newborn deaths occur within the first week of the postnatal period, it is the most neglected period [50]. Many women from low and middle-income countries and their newborns do not have access to health care during the early postnatal period [51].

Our analysis found that educational status has a significant effect across different quantiles of the CCI. Having completed primary and secondary education or above were associated with a higher COC than having no formal education, which is consistent with prior studies [30–32]. Maternal education is an important social determinant often linked to MCH and healthcare utilisation [52]. It enhances women’s decision-making power, economic independence, and empowerment, thereby increasing their access to and use of services [53].

Our results show that compared to women from the metropolis, women who were from Afar and Somali had a lower COC in all quantiles. At the 25th and 90th quantiles, women from Oromia also had a lower COC compared to women from the metropolis. Moreover, having been from Gambella and SNNPR were negatively associated with the COC at the lower quantile. Contrariwise, women from Tigray had a higher COC in the 10^th^, 50^th^, 75^th^, and 90^th^ quantiles compared with those who are from the metropolis. Women from Benshangul also had a higher COC at the 75^th^ quantile as compared to women from the metropolis. Variations in the completion of COC across the regions could be associated with geographical disparities in the country, such as accessibility, infrastructure availability, health service quality, women’s compliance with care and regional differences in socio-demographic characteristics, and religious or cultural beliefs. Individuals who live outside of metropolitan regions suffer from poor health outcomes attributed to multiple factors including less access to and use of care, financial hardships and disadvantages associated with fewer work prospects, and less educational attainment [54]. According to the EDHS report, Afar, Somali, and Oromia had the lowest coverage of most RMNCH services [41]. Another study showed that the use of RMNCH services in pastoralist regions of the country, such as Afar and Somali, and SNNPR is extremely low [55]. Ethiopia’s pastoral communities occupy 61% of the total landmass and 97% reside in the lowland areas of Afar, Somali, Oromia, Gambella, and SNNPR [56]. Often, pastoralists and semi-pastoralists live in marginal, remote, conflict-prone, and food insecure areas that experience higher morbidities and mortalities. Pastoralist populations have limited access to public services due to their mobile lifestyle and the limited infrastructure in remote areas. They also face complex barriers to healthcare access due to their deep sense of tradition, preference for self-treatment or traditional healers, and misconceptions about healthcare seeking, and some harmful traditional practices [57].

Consistent with previous studies [17,23], our findings indicate that living in a rural area was associated with low levels of the continuum at different quantiles. People from rural areas have limited access to health services due to distance, lack of transportation, and substandard facilities. Furthermore, poor access to media, comparatively high level of poverty coupled with strong cultural beliefs extensively render the lower completion of COC in rural areas [58]. The Health Extension Program is one of the most innovative community-based health programs launched in Ethiopia to make health services accessible to rural communities [59]. Despite the significant achievements in MCH, the program has been facing challenges related to the productivity and efficiency of health extension workers, working and living conditions of health extension workers, resource gaps, and lack of supportive supervision. To improve the program, health posts must be staffed with sufficient health workers having the right skills and motivation. Moreover, strengthening managerial supervision, allocating appropriate resources, and providing tailored intervention strategies play an important role in the successful implementation of the program [60].

At the median and highest quantiles, women from the poorest wealth quintiles had a lower COC compared to those from the richest. In line with our finding, a study indicated that the COC is low for women from the poorest households [28]. The low COC among the poorest women implies that the policies in place are not sufficient to compensate for the disadvantages associated with poverty. This is concerning, as health outcomes tend to be worse for those in the poorest and most vulnerable groups [61]. It has been argued that countries making the fastest progress towards coverage of MCH services were those that managed to reach the low socio-economic groups [62]. While MNCH services are provided for free in Ethiopia, economic gaps continue to contribute to observed discrepancies in healthcare utilisation [63]. Improving the COC among the poor requires coordinated action across different sectors so that women are educated and empowered, and poverty is reduced. Scaling up community-based services and primary healthcare facilities and implementing other pro-poor policies are also vital.

At the lowest and highest quantiles, mothers who did not perceive the distance to the nearest health facility as a problem had a higher COC compared to those who perceived the distance to the nearest health facility as a problem. Our finding is consistent with other studies [36,64]. Accessibility is an important factor in health service utilisation. Prior studies showed that long distance from home to health facilities coupled with poor transportation networks was associated with poor MCH outcomes [65–67].

Similar to other studies, we found that women of higher parity tended to have lower COC than those of lower parity (75th quantile) [25,26]. Women of higher parity may face challenges accessing services due to childcare responsibilities or resource constraints. These women may also rely on previous pregnancy experiences. It has been argued that as the number of children a mother has increases, the need to utilise healthcare services may fall. Women of lower parity have less pregnancy and childbirth experience and may have more desire to receive all the MCH services [68].

At the lower quantiles, women who didn’t intend the recent pregnancy had a lower COC compared to their counterparts. Studies showed that the completion of maternity care was higher among mothers whose pregnancy was intended [11,27]. Women with an unintended pregnancy may experience associated psychological issues such as stress and fear stigma from their partners or family members. Such women may conceal their pregnancies and become less motivated to utilise services. Moreover, problems related to work, education, or finances may prevent them from taking up MCH services [69].

In our study, caesarean delivery was positively associated with the COC at different quantiles. Likewise, a study reported that women who had caesarean delivery had a higher level of COC compared to their counterparts [28]. Such findings reinforce the hypothesis that increased risk perception encourages the use of essential services [70,71].

Our finding also re-affirms the noteworthy effect of early initiation of ANC visits for subsequent MCH services. Receiving ANC in less than four months of pregnancy was positively associated with COC across all quantiles. In line with this, a study stated that mothers who initiated ANC visit early had a higher COC than those who initiated late [11,35,72]. The timing of the first ANC contact is an important entry point to a continuation through the COC. It makes women better informed about pregnancy and the subsequent use of MCH services [73–75].

This study adds to the current literature by assessing the COC using a comprehensive approach incorporating a range of essential services. This is the first study, to our knowledge, that used quantile regression to explore the effects of variables at different points of the conditional distributions of CCI. Investigating the effect of covariates at different response quartiles is important as relationships may vary at different levels of the response. Quantile regression is a specific analysis used to determine whether relationships between a predictor and an outcome variable differ across levels of the outcome variable. It is an innovative method that provides a more nuanced interpretation of between-variable associations than the classical methods. Therefore, it is possible to identify the more vulnerable groups and devise more effective interventions.

The findings of this study clearly point to the need for targeted interventions that should adopt a multilevel approach to overcome the constraints in the COC. Effective public health interventions, especially strategic plans that stimulate PNC uptake for babies are essential. Given the effect of unintended pregnancy on the COC completion, early preventive strategies such as strengthening family planning services to assist women in having their desired number of children are required. In addition, more emphasis needs to be placed on the importance of early initiation of ANC. The findings also reinforce the need for new programmatic strategies that aim to reach illiterate and poor women. Moreover, government and other stakeholders should put more effort into scaling up the COC in rural areas and disadvantaged regions. Our study also highlights that improving women’s socio-economic position can promote the utilisation of MNCH services and hence improve the COC. Finally, strengthened collaborations between health and other sectors to increase access to health services may address critical leverage points in improving the COC.

This study has some limitations. DHS data are cross-sectional, and so cannot affirm any causal inference. In addition, recall bias is inherent in these surveys due to the nature of self-reported responses. However, our study population included women who had a live birth (most recent) in the two years preceding the survey.

## Conclusion

The COC is key to improving MNCH through integrated service delivery. CCI is an important indicator of the COC. We used a quantile regression model to identify the factors that affect the COC at different points of the conditional distributions of CCI. The overall completion rate of the COC was low, implying that women and children were not receiving the maximum possible health benefit from existing health services. PNC for newborns had the lowest coverage along the continuum. This study provides evidence that factors such as the educational status of women, region, residence, socio-economic status, perceived distance to a health facility, pregnancy intention, mode of delivery, parity, and early ANC initiation influence the COC differently across levels of the CCI. Understanding the predictors of the COC across the entire distribution of CCI will facilitate the development of targeted and focused evidence-based strategies to achieve better continuity of care and MNCH outcomes.
